# Primary Suture and Antiseptic Methods

**Published:** 1918-03

**Authors:** Cuthbert Wallace


### PRIMARY SUTURE AND ANTISEPTIC METHODS

General CUTHBERT WALLACE

Before I commence I should like to congratulate our French allies on the tremendous success they have had in their arrangements in Flanders, etc. They have set an example which I am sure we will follow, and which will result in great benefit to the wounded man.

Our trial of primary suture was arranged thus : — There were three clearing stations in the northern part of the army which had water evacuation to an advanced base. Advantage was taken of this to collect all cases which were primarily sutured and send them back to this place to be observed. I saw all the cases both at the front and at the base. Many were sent back to the base with their stitches still in. The journey was two miles on the ambulance, 12 hours on the barge and another mile on the ambulance, before they arrived at the hospital — an easy journey as ordinary evacuation goes, but involving a certain amount of disturbance. The number of the cases was 625, and of these 444 healed by first intention. 69 had unions with a certain amount of suppuration, and 69 were failures. The compound fractures are a striking series, because it shows that out of 100 fractures there were 75 o/o of successes. The proportion of cases on which primary suture was used was about 50 o/o of the wounded received.

You will see that various solutions were used.

A very successful experiment was made with the soap treatment, which, as a matter of fact, was first practised by French surgeons. 200 cases were thus treated with satisfactory results.

After primary suture there are a certain number of cases which give cause for thought. The skin gets tense and red, but though the temperature is high the pulse remains slow. From the clinical point of view the pulse-rate is a great criterion. It is better to trust the pulse-rate rather than the temperature in deciding whether to open the wound or not. The skin soon wrinkles, the swelling subsides, and the wound runs a perfectly good course.

Gas gangrene, to my mind, is not a bar to primary suture or at all events to delayed primary suture. The most striking successes we have had have been after gas gangrene. What is the cause of the success of primary suture?

No doubt a certain number of wounds in this war are not heavily infected. I have no doubt in my own mind that when the call for universal drainage came from the base its use actually led to the infection of a certain number of wounds owing to defective technique.

The principal factors are rapid evacuation and early surgical treatment. Those who have been a long time in France have had the advantage or disadvantage of tracing through many months and years the rise and fall of antiseptics. There is a danger that people coming out now for the first time and who have read about these different methods, may be apt to place more reliance upon the antiseptics than is justified. In the last three years the whole battle of antiseptics, dating from the time of Lister, has been fought out again.

I will give you very briefly the history of antiseptics in this war. It is really interesting and it may be beneficial. It has been said that this war has been a failure of antiseptics. As far as I know, antiseptics have been used to the exclusion of aseptics. In May 1915 the ordinary antiseptics of commerce were in full blast. This corresponds with the period when the wounded were dressed at the front and put on the train. Then came a period of drainage. At this time collapsible tubes with a convenient nozzle to introduce into a wound were sent to France. These collapsible tubes, filled with a greasy antiseptic, were given the surgeons working with regiments, and when a man was wounded the contents were squirted into his wounds. The treatment was stopped on account of the bad results obtained. It was obvious that unless the wounds were opened up the antiseptic did more harm than good. During 1915, later in the year, the hypochlorites were introduced, and a certain amount of improvement took place; but the surgery had progressed to a certain extent. Then we pass on to the next year. Early in 1916 we saw the rise of the Carrel method. As you all know, Carrel went through many experiments before this delightful and simple technique was evolved. The principle was the intermittent injection of a hypochlorite into the wound. At this time, too, the use of B. I. P. P. was coming into prominence. By 1916, at the battle of the Somme, excision was well understood and practised, and whether the B. I. P. P. or the Carrel method was used the wounded who had been operated upon arrived in good shape at the base. In the winter of 1916 and 1917 B. I. P. P. and Carrel ran side by side in the British army. Gradually B. I. P. P. ousted the Carrel method in the early treatment. Late in 1916 a small experiment on the suture of wounds was started. 1917 is remarkable for the rise of Flavine and those numerous colored pastes made up with a greasy base and an aniline dye. At the end of 1917 we see the excision of wounds coming in more and more.

I think now we have come to our senses. People talk about revolution in surgery. It is a return to sanity — a return to ordinary civil practice. You will find that the more war surgery approximates to civil surgery the better it will be. In civil life no one would be surprised if a man on the train with a compound fracture, operated on after 24 hours, did badly. The improvement that has taken place in the wounded is simply due to the fact that one now does what one was taught to do in the days of one's surgical infancy.

